# Editorial: Microbial diversity in mine drainage

**DOI:** 10.3389/fmicb.2026.1907976

**Published:** 2026-07-30

**Authors:** Ruiyong Zhang, Nancy Trun, Jun Liu, Michelle M. Valkanas

**Affiliations:** 1 State Key Laboratory of Advanced Marine Materials, Laboratory of Marine Environmental Corrosion and Bio-fouling, Institute of Oceanology, Chinese Academy of Sciences, Qingdao, China; 2 Bayer School of Natural and Environmental Sciences, Duquesne University, Pittsburgh, PA, United States; 3 National Key Laboratory of Agricultural Microbiology, College of Resources and Environment, Huazhong Agricultural University, Wuhan, China; 4 Pennsylvania Western University, California, PA, United States

**Keywords:** environmental impacts, geochemical parameters, microbial diversity, mine drainage, toxic pollutants

The mining industry is essential to the technological, economic, and infrastructural development of modern civilization. While mining is essential, the environmental challenges associated with these activities are of great concern (Hosseinpour et al., 2022). In particular, acid mine drainage (AMD) and mining-related drainage cause severe environmental impacts such as surface and ground water pollution, soil and habitat degradation, and aquatic ecosystem destruction (Akcil and Koldas, 2006; Shi et al., 2025; Pan et al., 2026). The chemical composition of mine drainage dictates the microbial diversity in these environments by drastically altering the physicochemical properties of the surrounding ecosystems (Huang et al., 2021; Ma et al., 2025). Pollution levels and environmental factors have a significant impact on the microbial community structure in mine drainage (Wei et al.). Mine drainage habitats serve as extreme ecological niches that support unique microbial diversity characterized by acidophilic, heavy metal-resistant, and sulfate-adapted microorganisms (Méndez-García et al., 2015; Munyai et al., 2021). Microorganisms that thrive in mine drainages are primary drivers of environmental degradation and potential keys to innovative remediation strategies (Levett et al., 2021; Oyetibo et al., 2021). Studying the diversity of microorganisms across acidic-to-neutral mine drainages, including microbial community interactions and their role in biochemical cycling, is key to transforming an environmental disturbance into a platform for sustainable bioremediation, resource recovery, and biotechnological advances Figure 1. The Research Topic “*Microbial diversity in mine drainage*” has received high-quality research connected to microbial diversity in various mine drainage systems and the impact of mine drainage on microbial communities. Seven articles have been published on this topic, including research and reviews, exploring the complex relationship between microbial life and the geochemical landscapes shaped by mining activities.

**Figure 1:**
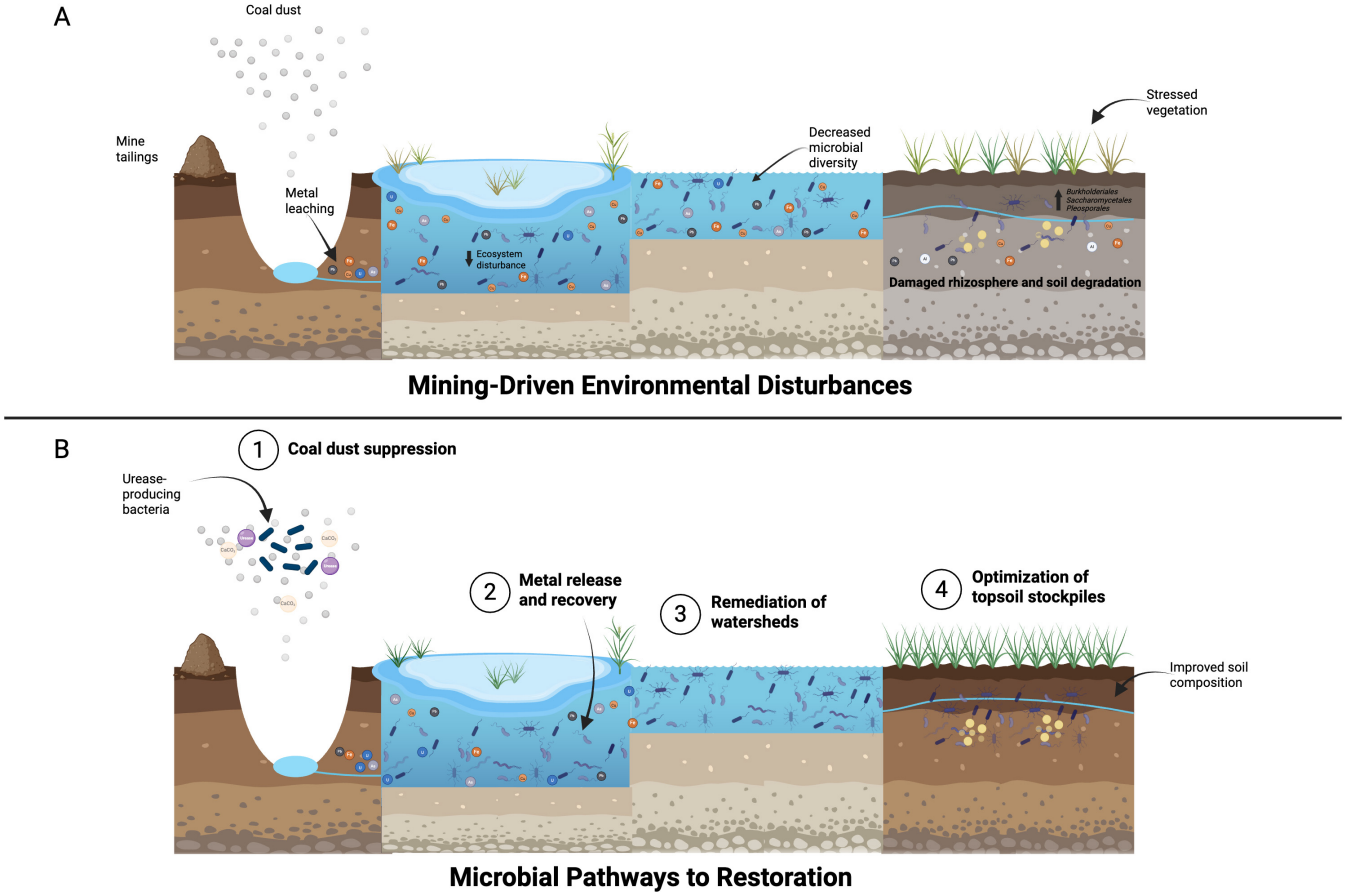
From environmental disturbance to microbially mediated restoration. Mining-driven environmental disturbances **(A)**. Microbial pathways to restoration **(B)**. Created in BioRender. Valkanas, M. (2026) https://BioRender.com/59188l7.

Understanding how microbial life survives in highly acidic mine drainage rich in heavy metals is crucial. Palomo et al. investigated the microbial diversity, community structure, and assembly mechanisms in AMD using high-resolution 16S rRNA gene amplicon sequencing with ecological modeling. The influence of environmental factors on microbial community composition was examined. Their work demonstrated that microbial communities survive in mine drainage by regulating their community assembly, niche partitioning, and function by driving the biogeochemistry of the mine drainage environment. Microbial communities are not merely passive inhabitants in mine drainage, but also active drivers of mineral transformation. It is crucial to understand the structure of microbial communities in the mine drainage system and the mechanisms by which microorganisms facilitate the release of metal ions. Wei et al. provided important insights into the biogeochemical release of uranium, showing how specific microbial communities facilitate the mobility of uranium. They investigated the influence of spatiotemporal variations on microbial community structure in water samples collected from various uranium-enriched acid mine drainage sites of an abandoned stone coalmine through geochemical and high-throughput sequencing analysis. Their study revealed the impact of uranium concentrations on microbial diversity with higher uranium concentrations reducing diversity. The variations in physicochemical parameters of samples also affected the structure of the bacterial community. Microbial communities such as acidophilic chemolithoautotrophic iron-oxidizing bacteria and acidophilic heterotrophic bacteria dominated at higher levels of uranium, whereas neutrophilic heterotrophic bacteria were more prevalent at low levels of uranium. Hagarová and Kupka used a metagenomic approach to analyze the microbiome and present a genetic map of metabolic pathways in microbial communities that are responsible for the geochemical transformation of Mária mine in RoŽnava, Slovakia. The microbial communities in the mine were dominated by Betaproteobacteria (>66%), consisting primarily of lithotrophic (mineral-consuming) organisms such as *Sulfuritalea* (6.93%), *Ferrigenium* (5.45%), *Gallionella* (3.79%), and *Sideroxydans* (3.65%), alongside the heterotrophic genus *Pseudomonas* (5.2%). The metagenomics data revealed key metabolic drivers such as abundant genes for iron uptake (TC.FEV.OM), the Sox enzyme system for sulfur oxidation, and the RubisCO enzyme for CO_2_ fixation. Resistome profiling revealed a strong correlation between microbial resistome and the mineralogy of the mine.

The impacts of mine drainage extend far beyond the drainage itself, affecting the geochemistry and microbial communities of the surrounding ecosystems. Analyzing the mine drainage impact on geochemical parameters and microbial communities of local ecosystems helps to understand the interaction between them and provides a foundation for more effective ecosystem management and treatment. Wan et al. studied how iron ore mining affects the rhizosphere fungal communities of local flora by examining their correlation with physicochemical properties and heavy metal concentration levels in rhizosphere soil. Their study showed that iron ore mining affected the physical and chemical properties of rhizosphere soil and its associated fungal communities. Toxic substances from mine drainage affect the structure and assembly of microbial communities. Understanding the structure and assembly of microbial communities in mine drainages can aid in understanding their survival in various levels of toxic pollutants, as well as which microbial groups cause or mitigate environmental pollution. Li et al. elucidated how microbial communities, particularly bacterial and fungal communities, shape their composition, co-occurrence networks, and assembly process depending on the levels of nutrients and heavy metals present in tailings soil and biological soil crusts (BSCs). Their findings revealed that physicochemical properties, microbial diversity, community structure, and ecological interactions varied according to levels of pollution. Under high pollution levels, microbial diversity decreased and showed a strong correlation of bacterial and fungal communities with environmental factors. In particular, under high pollution, bacterial communities' assembly followed a deterministic process, whereas fungal assembly remained largely stochastic. Research by Fischer et al. investigated how the physical handling of topsoil stockpiles can drastically alter the viability of the microbial community, providing insights for restoration. Their findings revealed that microbial diversity decreased and microbial community structure changed with depth in the stockpiles. Geochemical factors such as phosphorus and boron availability, as well as changes in organic matter, were the main driving forces in shaping the microbial communities within the stockpiles.

Dust or particulate matter (PM) released during mining operations is a major concern for human health and the environment. Various techniques are used to reduce and control mining dust, such as water spraying, the installation of enclosed rooms with air filtration and proper ventilation systems, and the use of dust suppression chemicals. These techniques often consume large amounts of energy and can generate secondary pollutants. Tastambek et al. discussed the use of microbial-induced carbonate precipitation as a sustainable biotechnological solution for environmental issues in the coal industry, particularly coal dust suppression. Their work provides a detailed account of how microbes prevent dust from spreading in the air and the mechanisms underlying this process. Specifically, urease-producing bacteria promote the formation of calcium carbonate (CaCO_3_) that binds the fine coal particles, creating a stable crust that improves surface stability and prevents the dispersion of airborne dust. Strategies such as synthetic biology and waste-to-resource can make the microbial-induced carbonate precipitation process economically viable for mitigating dust or particulate matter released during mining operations in the future.

The articles published on this topic underscore that microbial communities present in mine drainage act as natural chemical engineers, controlling the health of the surrounding environment, and offer untapped potential for green technology innovation. Therefore, studying the microbial communities in mine drainage is of great importance for their application in environmental remediation and metallurgical applications (Palomo et al.). Maximizing the efficacy of microbial communities in environmental remediation and metallurgical applications necessitates a comprehensive understanding of diversity, structure, assembly of microbes, and their interaction with the geochemistry and mineralogy of mine drainage (Peng et al., 2025a). The extent to which mine drainage affects the geochemical properties and microbial communities in the surrounding areas continues to be investigated (Peng et al., 2025b; Zhang et al., 2025). The results of published work on this topic highlight that future research should focus on long-term monitoring to ensure that changes in geochemical properties do not trigger secondary pollution by microbial communities. It highlights the need for extensive research into the effective use of microorganisms to control mine dust or particulate matter, thereby improving mine safety and air quality.

## References

[B1] Akcil A. Koldas S. (2006). Acid Mine Drainage (AMD): causes, treatment and case studies. J. Clean. Prod. 14, 1139–1145. doi: 10.1016/j.jclepro.2004.09.006

[B2] Hosseinpour M. Osanloo M. Azimi Y. (2022). Evaluation of positive and negative impacts of mining on sustainable development by a semi-quantitative method. J. Clean. Prod. 366:132955. doi: 10.1016/j.jclepro.2022.132955

[B3] Huang Y. Li X.-T. Jiang Z. Liang Z.-L. Wang P. Liu Z.-H. . (2021). Key factors governing microbial community in extremely acidic mine drainage (pH < 3). Front. Microbiol. 12:761579. doi: 10.3389/fmicb.2021.761579 34917049 PMC8670003

[B4] Levett A. Gleeson S. A. Kallmeyer J. (2021). From exploration to remediation: a microbial perspective for innovation in mining. Earth-Sci. Rev. 216:103563. doi: 10.1016/j.earscirev.2021.103563

[B5] Ma R. Kong J. Mou H. Liu B. (2025). Mine drainage leads to the reshaping of the spatial patterns of soil extracellular econzymatic stoichiometry and microbial resource limitation. Sci. Rep. 15:42844. doi: 10.1038/s41598-025-20858-1 41309647 PMC12669616

[B6] Méndez-García C. Peláez A. I. Mesa V. Sánchez J. Golyshina O. V. Ferrer M. (2015). Microbial diversity and metabolic networks in acid mine drainage habitats. Front. Microbiol. 6:475. doi: 10.3389/fmicb.2015.00475 26074887 PMC4448039

[B7] Munyai R. Ogola H. J. O. Modise D. M. (2021). Microbial community diversity dynamics in acid mine drainage and acid mine drainage-polluted soils: implication on mining water irrigation agricultural sustainability. Front. Sustain. Food Syst. 5:701870. doi: 10.3389/fsufs.2021.701870

[B8] Oyetibo G. O. Enahoro J. A. Ikwubuzo C. A. Ukwuoma C. S. (2021). Microbiome of highly polluted coal mine drainage from Onyeama, Nigeria, and its potential for sequestrating toxic heavy metals. Sci. Rep. 11:17496. doi: 10.1038/s41598-021-96899-z 34471151 PMC8410811

[B9] Pan Y. Fang Z. Chen Y. Zhang J. Lu G. Dang Z. . (2026). Clean water irrigation promotes microbial community recovery in acid mine drainage-contaminated paddy soil: a spatiotemporal analysis based on simulated soil column experiments from Dabaoshan mine, China. Environ. Sci. Process. Impacts 28, 74–83. doi: 10.1039/D5EM00762C 41431229

[B10] Peng F. Wu B. Zheng Q. Long Y. Jiang J. Hu X. (2025a). Long-abandoned mining environments reshape soil microbial co-occurrence patterns, community assembly, and biogeochemistry. Appl. Soil Ecol. 213:106254. doi: 10.1016/j.apsoil.2025.106254

[B11] Peng S. Bao N. Zhao X. Meng D. Han Z. (2025b). How soil physicochemical properties and microorganisms change under mining activities in alpine and high-altitude regions–a case study of a copper mine area in Tibet Plateau. J. Environ. Chem. Eng. 13, 117202. doi: 10.1016/j.jece.2025.117202

[B12] Shi X. Gao Y. Qian H. Chen J. Li W. Li S. . (2025). Elucidating the hydrochemistry and REE evolution of surface water and groundwater affected by acid mine drainage. Environ. Pollut. 366:125495. doi: 10.1016/j.envpol.2024.125495 39647767

[B13] Zhang S. Wu P. Zhang J. Li Q. Luo C. (2025). Effects of acid mine drainage on microbial community development and physicochemical properties of mine contaminated sites in Southwest China. Sci. Rep. 15:20776. doi: 10.1038/s41598-025-05799-z 40594805 PMC12218272

